# Supplementary material for: Which Is the Best Treatment in Recurrent Thymoma? A Systematic Review and Meta-Analysis
Source: Cancers (Basel). 2021 Mar 29;13(7):1559. doi: 10.3390/cancers13071559 (PMC8036834; doi:10.3390/cancers13071559)
Supplement: Supplementary file 1 [file cancers-13-01559-s001.pdf]

Table S1. The quality assessment of the included studies.

STUDY	*Q1*	*Q2*	*Q3*	*Q4*	*Q5*	*Q6*	*Q7*	*Q8*	*Q9*	*Q10*	*Q11*	*total*
RUFFINI (1997)	*Y*	*Y*	*Y*	*N*	*N*	*Y*	*Y*	*U*	*N*	*N*	*Y*	*6*
MARGARITORA (2011)	*Y*	*Y*	*Y*	*Y*	*U*	*Y*	*Y*	*Y*	*Y*	*NA*	*Y*	*9*
HAMAJI (2012)	*Y*	*Y*	*Y*	*Y*	*Y*	*Y*	*Y*	*Y*	*Y*	*Y*	*Y*	*11*
BAE (2012)	*N*	*Y*	*Y*	*Y*	*U*	*Y*	*Y*	*N*	*N*	*N*	*Y*	*6*
SANDRI (2014)	*Y*	*Y*	*Y*	*Y*	*Y*	*Y*	*Y*	*Y*	*N*	*N*	*Y*	*9*
MIZUNO (2015)	*N*	*Y*	*Y*	*Y*	*Y*	*U*	*Y*	*Y*	*Y*	*N*	*Y*	*8*
MARULLI (2016)	*N*	*Y*	*Y*	*Y*	*Y*	*Y*	*Y*	*Y*	*N*	*N*	*Y*	*8*
FIORELLI (2017)	*Y*	*Y*	*Y*	*Y*	*Y*	*Y*	*Y*	*Y*	*N*	*N*	*Y*	*9*
CHIAPPETTA (2019)	*Y*	*Y*	*Y*	*Y*	*Y*	*Y*	*Y*	*Y*	*N*	*N*	*Y*	*9*

Y: Yes; N: No; U: Unclear; NA: Not Applicable .

Questions:
1. Were the two groups similar and recruited from the same population?
2. Were the exposures measured similarly to assign people to both exposed and unexposed groups?
3. Was the exposure measured in a valid and reliable way?
4. Were confounding factors identified?
5. Were strategies to deal with confounding factors stated?
6. Were the groups/participants free of the outcome at the start of the study (or at the moment of exposure)? 7. Were the outcomes measured in a valid and reliable way?
8. Was the follow up time reported and sufficient to be long enough for outcomes to occur?
9. Was follow up complete, and if not, were the reasons to loss to follow up described and explored?
10. Were strategies to address incomplete follow up utilized?
11. Was appropriate statistical analysis used?

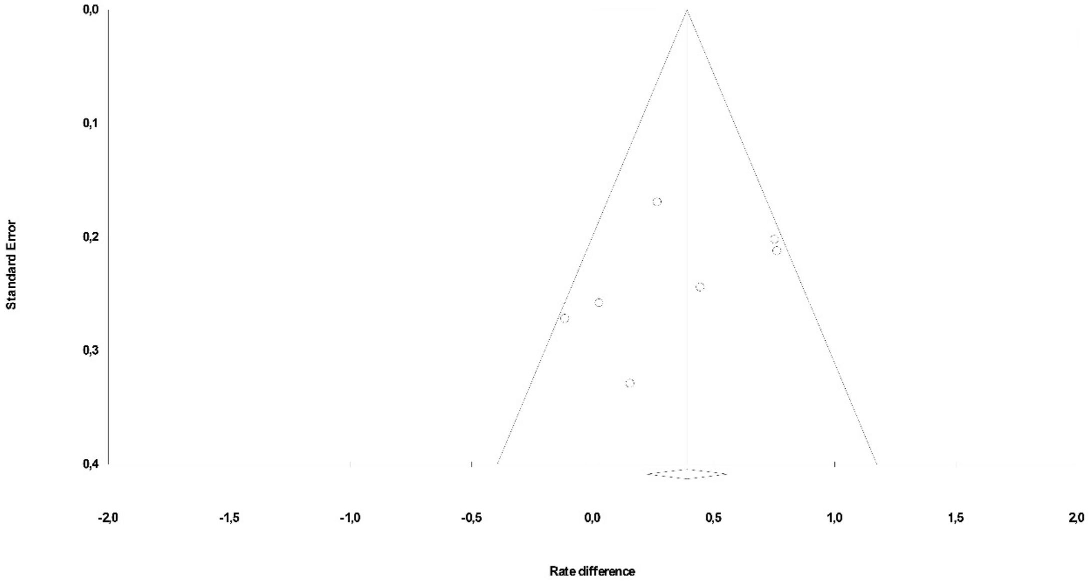

Figure S1. Funnel Plot for standard error for surgery versus other treatments
